# Prevalence of Dunning-Kruger effect in first semester medical students: a correlational study of self-assessment and actual academic performance

**DOI:** 10.1186/s12909-024-06121-7

**Published:** 2024-10-24

**Authors:** Harald Knof, Markus Berndt, Thomas Shiozawa

**Affiliations:** 1 Department of Anatomy, Institute of Clinical Anatomy and Cell Analysis, Faculty of Medicine, Eberhard Karls University of Tuebingen, Elfriede-Aulhorn- Straße 8, 72076 Tuebingen, Germany; 2 Institute of Medical Education, LMU University Hospital, LMU Munich, Pettenkoferstraße 8a, 80336 Munich, Germany

**Keywords:** Dunning-Kruger effect, Self-assessment, Medical student, Self-regulation, Health professions education, Educational Measurement, Medical education

## Abstract

**Introduction:**

The ability to self-assess is a crucial skill in identifying one’s own strengths and weaknesses and in coordinating self-directed learning. The Dunning-Kruger effect occurs when limited knowledge causes individuals to overestimate their competence and underestimate others’, leading to poor self-assessment and unrecognized incompetence. To serve as a foundation for developing strategies to improve self-assessment, the self-assessment abilities of first-semester students were assessed.

**Methods:**

In the final weeks of the summer 2021, winter 2021/22, and summer 2022 semesters, the academic performance (oral anatomy exam) of first semester students was assessed (0–15 points). Before the exam results were announced, students were asked to self-assess their performance.

**Results:**

Exam scores (*M* = 10.64, *SD* = 2.95) and self-assessed scores (*M* = 10.38, *SD* = 2.54) were comparable. The absolute difference between them, as a measure of self-assessment ability ranged from − 9 to + 9 points (*M* = -0.26, *SD* = 2.59). Among participants (*N* = 426), 18.5% assessed themselves accurately, 35.5% overestimated, and 46.0% underestimated their performance. The correlation between actual score and self-assessment was ρ = -0.590 (*p* < 0.001), reflecting the Dunning-Kruger effect. When separated by gender, correlation for females was ρ = -0.591 (*p* < 0.001), and for males ρ = -0.580 (*p* < 0.001).

**Conclusions:**

Realistic self-assessment is a challenge for first-semester students. The data indicate that females tend to overestimate their performance while males underestimate theirs. A pronounced Dunning-Kruger effect is evident in both genders, with significant negative correlations between self-assessment and actual performance. There are several reasons for the occurrence of the Dunning-Kruger effect. Considering that the COVID-19 pandemic influenced learning environments, collaborative learning was significantly restricted. The lack of opportunities for comparison could potentially lead to unrealistic self-assessment.

## Introduction

Self-assessment and self-assessment ability are important skills for medical students, they are essential to self-directed, life-long learning and the practice of medicine [1–4]. As part of professional self-regulation, self-assessment can help in identifying weaknesses and strengths, enhancing self-directed learning activities, self-improvement, and professional development [5–8]. To enhance performance during learning, students need to identify what they must learn, while simultaneously advancing with confidence becomes much easier when one is aware of one´s strengths [8]. In areas of limited competence, self-assessment allows to self-limit plans of actions or to recruit additional resources. This is a crucial aspect when talking about patient safety [4, 8, 9]. Self-assessment is often inaccurate and overestimation and underestimation may occur [10]. This is important to know, because only realistic self-assessment can shape realistic expectations, appropriate developmental learning goals, and be a stimulus to further learning [5, 9, 10]. It should be noted that the format of the task does not influence students’ abilities to assess their own performance [4]. To foster and augment authentic self-assessment, students should cultivate metacognitive abilities and engage in systematic reflection upon their learning experiences [11].

Several studies have shown that self-assessments by medical students and physicians in different career steps can be invalid measures of their objective performance [5, 12, 13]. It has been shown that students who don’t perform well on assessments typically overestimate their performance. Students who perform very well are more likely to assess themselves accurately or even underestimate their performance [11, 13, 14]. This instance exemplifies the educational psychological phenomenon identified as the *Dunning-Kruger effect*. The authors posit that underperforming students not only exhibit a deficiency in content knowledge but also lack the metacognitive capacity necessary for discerning inadequacies in their content knowledge. Consequently, these students tend to overestimate their performance [15].

At the so-called “Mount Stupid”, unexperienced people often mistakenly overestimate their performance; they don’t know what they don’t know [12]. As time and experience grows, one realizes that one was unaware of some of the more complicated aspects of the issue. Then one falls into the “Valley of Despair”, and self-confidence is shattered. If time and energy are continuously put into the work, more experience and wisdom is acquired, subsequently enabling to reach the “Slope of Enlightenment” and the “Plateau of Sustainability”.

The Dunning-Kruger effect even influences the quality of medical education, as it affects the decision-making ability in cases of studying, self-directed learning, and thus the learning curve of each individual student [4, 13].

The previously described Dunning-Kruger effect occurs in various learning situations and when assessing different parameters, as demonstrated in numerous studies [16–18]. Some studies focused on the mere prediction of scores, but this effect can also be observed in the prediction of the percentile rank in which a score lies [15, 19–23].

When examining students from different academic disciplines, students in chemistry and physics, medicine, dentistry, business administration, and management, as well as aviation or nursing are affected by the Dunning-Kruger effect [11, 13, 14, 24–27].

Further research has shown the presence of the Dunning-Kruger effect when investigating simple tasks, such as pure knowledge tests [28, 29], as well as more complex skills like driving [30], debating performance [31], reading [32], or coaching ability in volleyball [33].

Even in different medical procedures like central venous catheterization, basic life support, ECG-recording and ultrasound [26, 34, 35], or complex clinical processes such as disease diagnosis, surgical procedures, or performance in anesthesiology and pathology residency training programs, a discrepancy between confidence and competence can be observed [17, 36–39].

It appears that neither the nature of the task, nor the level of training, specialty, the domain of self-assessment, or manner of comparison does influence the accuracy of self-assessments, although it is suspected that overestimation is more likely during performance situations involving more subjective interactions and evaluations [4, 10, 17].

The timing of self-assessment appears to play a minor role in its accuracy. On the one hand, students were more accurate in their self-assessments after completing a task and when both the task and criteria were objectively measured [10]. On the other hand, the Dunning-Kruger effect was also observed when students predicted their performance before completing an assessment [11, 27]. Meta-analyses have shown that students’ self-assessment accuracy improves in later years of medical school, as their evaluations become more self-critical over time. However, even at advanced career levels, the correlation between self-assessment ability and actual performance remains limited [5, 8, 10, 17].

Most studies recognize the existence of the Dunning-Kruger effect and provide psychological explanations, sometimes agreeing with and sometimes challenging Kruger and Dunning’s metacognitive explanation that low performers overestimate their abilities because they are unaware of how poorly they performed due to their lack of knowledge [40–43]. Statistical explanations for the Dunning-Kruger effect include the hard-easy effect, the regression to the mean effect, the “better than average” (BTA) approach, the influence of measurement errors, and boundary restrictions [18, 22, 23, 42, 44–47]. Some authors suggest that the psychological processes involved in self-assessment may be, to some extent, consciously motivated and/or related to personality traits [48]. The Dunning-Kruger effect could also be explained using the anchoring and adjustment heuristics [16]. It has been shown that people who self-assessed their intelligence, on average, rated themselves five IQ points lower after completing an intelligence test, compared to those who self-assessed their IQ before taking the test [49]. It was also found that poor test performers overestimated their results significantly less when they were rewarded for the accuracy of their self-assessment [50]. Traits such as narcissism have also been identified as predictors of discrepancies between self-assessed and objectively measured intelligence [51, 52].

However, recent literature underscores the metacognitive explanation of the Dunning-Kruger effect as proposed by Dunning and Kruger [18, 41, 53].

Medical students who have successfully navigated the challenging admission process for medical school, which requires an excellent high school diploma, tend to make optimistic performance predictions [5, 54]. These optimistic predictions are largely attributed to the strong correlation between high performance expectations and a history of academic success [54, 55]. This relationship suggests that students’ positive self-assessments, shaped by their past achievements, reinforce their confidence in future performance, regardless of potential gaps in actual knowledge or ability [54].

In a study on learning strategies among first-semester medical students, it was demonstrated that changes in learning conditions due to COVID-19, as well as the varied use of collaborative learning strategies, impact first-semester students’ self-assessment abilities [56]. However, this initial study only examined a small cohort. To gain a more comprehensive understanding of the self-assessment skills of first-semester students, a larger study population was necessary. Therefore, the authors included two additional student cohorts for this study to investigate the occurrence of the Dunning-Kruger effect. The first academic semester represents a crucial period for examining students’ self-assessment abilities.

As first-year medical students usually are at the beginning of their learning curve, possible interventions to improve self-assessment can be most effective. But it is known that adequate calibration among low performers is unlikely to happen by itself [18].

The first step, however, is to analyze the extent of current self-assessment ability, as a foundation for the development of strategies to improve self-assessment.

Hence, the aim of this research is to evaluate the prevalence of the Dunning-Kruger effect in first-year medical students.

On this basis, strategies can be developed to enhance self-assessment, promote self-directed learning, elevate learning curves, and contribute to the academic success of students.

The following hypotheses were proposed:

H_1_: There are discrepancies between self-assessment and actual exam performance, consistent with the Dunning-Kruger effect, where objectively low-performing students tend to overestimate their abilities, while high-performing students tend to underestimate their abilities.

H_0_: There are no discrepancies between self-assessment and actual exam performance, consistent with the Dunning-Kruger effect, where objectively low-performing students tend to overestimate their abilities, while high-performing students tend to underestimate their abilities.

## Methods

### Curriculum

In the first semester of the medical program at the investigated university students receive a 64-hour introductory lecture of anatomy, covering major topics for a general overview of the field. The lecture content is assessed with an oral examination at the end of the semester.

### Data collection

Medical students of summer semester 2021, winter semester 2021/22, and summer semester 2022 were invited to participate in a survey and self-assessment of their academic performance. At this point, the participants had completed all courses of the first semester of their medical study program. Students were invited to participate by the first author in person. For their participation, the students received an expense allowance of €5. Written informed consent was obtained from all participants prior to the study. They were informed about the chances, risks, rights, obligations, and the voluntariness of the investigation. Data were collected in pseudonymized form. All participants also agreed to the publication of the data in anonymized form. Students could revoke their consent without incurring any disadvantage. Ethical approval for this study was obtained from the ethics committee at Eberhard Karls University Tuebingen with letter no. 086/2022BO2.

## Measures

### Examination performance

The oral examination of the introductory anatomy lecture was conducted in a structured viva, with a time limit to 6 min [57]. The examiners were academic staff, students had no chance to interact with each other before completion of the viva examination due to an one-way-parkour. As each of the examiners lectures one part, they create matching open-ended questions on the respective topic. These question catalogues are also accessible to the students and can be used both by the examiners to draw exam questions, and by the students to prepare for the exam. To differentiate the performance in the oral examination, the grade point system of the German senior years of secondary school was used. The grading scale is as follows: *fail* for a score of zero, *sufficient* for scores one to three, *satisfactory* for scores four to six, *good* for scores seven to nine, *very good* for scores 10 to 12, and *excellent* for scores 13 to 15. Grades below *satisfactory* are considered a failure, while grades from *satisfactory* and above are considered a pass.

### Subjective self-assessment

In conjunction with a questionnaire survey on collaborative learning and learning organization [56], participants were also asked to evaluate their performance in the oral examination prior to the official announcement with one item (*“For the oral examination in anatomy*,* I would have given myself the following grade: __“)*. This self-assessment used the same grade point system as described above. The difference between the self-assessed score and the actual score represents the self-assessment ability. Positive deviation marks overestimation, negative deviation marks underestimation, respectively. The greater the deviation, the lower the grade of realistic self-assessment ability.

### Data analysis

After a descriptive analysis, correlations according to Spearman were performed. Significance of the obtained results was judged at the 5% level. For collecting questionnaire responses, extraction, and analyses, SPSS Statistics 29 (IBM Corp., Armonk, NY) was used.

## Results

A total of *N* = 490 first-semester medical students were surveyed using a paper-based questionnaire. With a total admission of 210 students per semester, a maximum of 630 participants could have been included; this represents a response rate of 77.8%. Participants with any missing data were excluded. This resulted in the final sample of *N* = 426 respondents.

Study participants were 71.5% female, this corresponds to the gender distribution of the admission. The age ranged from 18 to 42 years; the mean age was 20.87 years (*SD* 2.85). Most participants were medical students (417 = 97.4%) followed by dentistry students (10 = 2.3%) and one student of molecular medicine (0.2%). The allocation of study places in the first semester is structured as follows: 30% of the places are reserved for applicants with the best high school grades, 10% are allocated through the *additional aptitude quota* which includes criteria such as work experience in a healthcare-related professions, and 60% are distributed via the selection process of the universities. Additionally, 5% of the study places are reserved for non-EU foreign students, who can apply directly to the faculties. Although specific data on this demographic distribution was not explicitly collected, it can be assumed, given the high response rate, that the distribution in the present sample is similar.

The scores obtained in the anatomy exam range from 4 to 15 points (*M* = 10.64, *SD* = 2.95) and the self-assessed score for the anatomy exam from 1 to 15 points (*M* = 10.38, *SD* = 2.55). The absolute difference between the self-assessed score and the actual score as a measure of self-assessment ability range from − 9 (underestimation) to + 9 points (overestimation) (*M* = -0.26, *SD* = 2.59). Underestimation was observed more often than overestimation. The proportion of women overestimating themselves was greater than the proportion of men overestimating themselves (Table 1).

**Table 1:** Correct self-assessment, over- and underestimation by genders and semester

	Female students						Male students					
	Underestimation		Correct self-assessment		Overestimation		Underestimation		Correct self-assessment		Overestimation	
	Frq.	Pct.	Frq.	Pct.	Frq.	Pct.	Frq.	Pct.	Frq.	Pct.	Frq.	Pct.
summer semester 2021	31	38.8%	20	25.0%	29	36.3%	16	59.3%	4	14.8%	7	25.9%
winter semester 2021/22	50	47.2%	17	16.0%	39	36.8%	30	57.7%	7	13.5%	15	28.8%
summer semester 2022	52	43.3%	23	19.2%	45	37.5%	18	43.9%	7	17.1%	16	39.0%
Total	133	43.5%	60	19.6%	113	36.9%	64	53.3%	18	15.0%	38	31.7%

(Frq. = Frequency; Pct. = Percent)

**Fig. 1:**
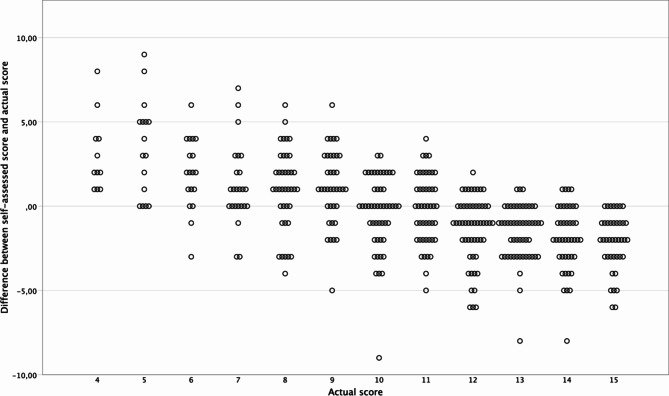
Scatterplot of realistic self-assessment (difference between self-assessed score and actual score) and actual score

An analysis according to Spearman was carried out to examine the relationship between the actual score and the difference between self-assessed score and actual score as a measure of self-assessment ability. The correlation was ρ = -0.590 (*p* < 0.001); separated by gender for females ρ = -0.591 (*p* < 0.001) and for males ρ = -0.580 (*p* < 0.001), indicating that the higher the overestimation, the lower the exam performance and the higher the underestimation the higher the actual exam performance (Fig. 1).

When analyzing the data, the gender differences led to further investigation. It is noticeable that in all semester cohorts studied, the percentage of female students assessing themselves realistically is higher than the percentage of their male peers.

In summer semester 2021 and winter semester 2021/22, more male students underestimate themselves than female students, who tend to overestimate themselves. In summer semester 2022, more male students overestimate themselves than female students.

This also leads to gender differences in correlation coefficients (Table 2).

**Table 2:** Correlation coefficients between the score received and self-assessment ability by gender and semester

	Female students		Male students	
	ρ	*p*	ρ	*p*
summer semester 2021	-0.638	< 0.001	-0.408	0.034
winter semester 2021/22	-0.582	< 0.001	-0.542	< 0.001
summer semester 2022	-0.560	< 0.001	-0.711	< 0.001

## Discussion

In the introduction, the frequent occurrence of overestimation and underestimation of one’s own performance was described. Additionally, the Dunning Kruger effect and its prevalence were explicated.

In the present study, instances of overestimation and underestimation manifest more frequently than realistic assessments of examination performance. Notably, the percentage of female students engaging in realistic self-assessment surpasses that of their male peers. The Dunning-Kruger effect can be observed, as a robust negative correlation exists between unrealistic self-assessment, particularly overestimation, and diminished exam performance.

First, the general occurrence of the Dunning-Kruger effect will be discussed, followed by the background and explanations of semester differences. Looking at Fig. 1, it is noticeable that there are no students who did not pass the exam listed. The respective students immediately received information about their non-passing status and were therefore excluded from the study, as they were unable to make an independent self-assessment. Moreover, it must be considered as a limitation that overestimation in higher grade ranges is limited by the scale itself. This implies that students can overestimate less in the higher point range than in the lower point range. These so-called “bounded data” play a critical role in understanding why people misjudge their abilities. The constraints in the data (both actual performance and self-assessment scores) naturally lead to overestimation by lower performers and underestimation by higher performers, as students in the bottom quartile can only make optimistic errors placing themselves into a higher quartile, while students in the top quartile can only make pessimistic errors placing themselves in a lower quartile [40, 42].

There are several reasons for inaccurate self-assessment, which can lead to the occurrence of the Dunning-Kruger effect [2]. It is possible that ambitious students set stricter standards for themselves and evaluate themselves against their potential and not on their actual performance [5, 54].

The literature suggests that students often evaluate themselves based on effort rather than performance, leading to significant disparities in self-assessment accuracy, with weaker students tending to overestimate their abilities and stronger students underestimating themselves to a lesser degree [54, 58, 59].

There may also be a certain amount of self-deception, as most medical students have done well in school and received strong positive feedback from an early age on. As a result, they have a strong sense of self-confidence that may be resistant to change [54]. Overestimation can also be used as a defense mechanism to compensate for poor performance, especially in in conjunction with the shame of admitting underperformance [54, 60].

Below-average students tend to push their own performance towards the average or passing grade by overestimating it – the “better-than-average-approach”, while high-performers weaken their performance because they tend to glorify the mistakes they made in the exam [47, 60]. On average, most people believe they are better or perform better than others [22, 23, 47, 61]. Despite this belief being irrational since “it is logically impossible for most people to be better than the average person” (Taylor & Brown, 1988, p. 195) [61]. This bias reflects the broader pattern of illusory superiority, where individuals inaccurately perceive themselves as being above average, distorting objective self-assessments.

Another possible explanation for this effect is the hard-easy effect [46]. The hard-easy effect occurs when difficulty is measured by the percentage of correct answers; in such cases, as the difficulty of the questions increases, so does the degree of overconfidence [45]. Regardless of the testing environment, which is objectively equally for all participants, the subjective difficulty varies. Thus, the test is difficult for those with low scores but easy for those with high scores, and this subjective difficulty drives overestimation [46].

As a result, self-assessment is distorted by conscious and unconscious intrinsic bias factors [60]. The phenomenon of social desirability also contributes to the fact that self-assessment of performance is not realistic [60]. Students have a tendency to think that they always tend to be more embarrassed than others when an unfavorable self-assessment is given [62]. This does not take into consideration that fellow students may also have the same feeling. In their self-assessment, they try to be more self-confident or socially desirable and therefore rate themselves unrealistically [60].

Metacognitive skills essential for realistic self-assessment, developed through self-reflection and external feedback, underpin an individual’s ability to evaluate and enhance their competence objectively. In parallel, feedback literacy, as conceptualized by Molloy et al. (2019), involves a nuanced understanding of feedback dynamics, emphasizing effective comprehension, constructive delivery, and fostering a culture of continuous learning and improvement through clear and respectful communication. Together, these competencies form a holistic framework for informed self-evaluation and sustained personal and professional growth [63]. Since the sample consists of students in their first semester, these metacognitive skills must first be developed in the setting of university teaching [15, 24, 64]. It is known that that medical students’ self-assessment becomes more self-critical over time and less prone to overestimation [5, 8].

Kruger and Dunning (1999) mentioned in their initial discussion of the effect that there could be boundary conditions, in the form of a minimum threshold of learning or experience, that must be crossed before excessive confidence in poor performance is displayed [15]. It has been shown that even a little learning experience could lead individuals into a situation where they become some of the most susceptible to the Dunning-Kruger effect leading them to overestimate their performance inappropriately [65]. The first-year cohorts observed in this study undoubtedly belong to exactly this group. Thus, it’s not surprising that the Dunning-Kruger effect manifests.

What is striking, is the manifestation of the Dunning-Kruger effect across the different surveyed semester cohorts and genders. In contrast to literature [10], our data show that female students overestimate themselves more than male students. While in the summer semester 2021 and winter semester 2021/22 the prevalence of overestimation among female students is greater than among male colleagues, in the summer semester 2022 it is the other way around.

It can be discussed if the learning environments altered by the COVID-19 pandemic may have had an influence on self-assessment behavior [56]. In the summer semester of 2021, teaching took place fully digitally and collaborative learning was significantly restricted. Normally, first-semester students concentrate on establishing personal relationships and cultivating a professional network pertinent to their studies [66]. During the COVID-19 pandemic in Summer 2021, the conditions to form personal relationships and study groups were not given. Social integration and personal interaction were more difficult, as contact with fellow students was only possible digitally [67, 68]. All this hampers the formation of learning groups or other collaborative learning formats. When collaborative learning occurs, students can compare with other group members, the knowledge levels of other learners can be related to one’s own, and thus students are able to develop a (social) reference standard. This standard helps them to assess themselves adequately. The lack of comparison with other students, combined with the restriction of private activities due to COVID-19 regulations, may have contributed to students spending more time on learning activities, due to a lack of alternative leisure options and assuming that other students would also be studying. Having immersed themselves in the content extensively, individuals may experience a sense of confidence that can result in overestimation. Both in winter semester 2021/22 and in summer semester 2022, collaborative learning opportunities were increasingly created again, and students were able to interact more closely and compare levels of knowledge with each other. An indication of this is the increase in realistic self-assessments, especially among male students. But the question remains as to what causes the discrepancy between male and female students.

## Conclusion

Realistic self-assessment is a major difficulty for students at the beginning of their studies. From our data we can conclude that women tend to overestimate their performance and male students underestimate themselves. This shows a pronounced Dunning-Kruger effect, with significant negative correlations between self-assessment and actual performance. The data must be seen in the light of the then present COVID-19 conditions.

Further research should address how self-assessment skills can be taught at the beginning of medical studies to counteract these findings. Additionally, there is a need for further investigation into the underlying reasons for gender-specific differences in self-assessment within the academic setting.

## Data Availability

The datasets used and analysed during the current study are available from the corresponding author on reasonable request.
